# Prognosis of patients with acute respiratory failure due to the SARS-CoV-2 501Y.V2 variant: a multicenter retrospective matched cohort study

**DOI:** 10.1038/s41598-022-12767-4

**Published:** 2022-05-24

**Authors:** Bérénice Puech, Antoine Legrand, Olivier Simon, Chloé Combe, Marie-Christine Jaffar-Bandjee, Margot Caron, Charles Vidal, Patrick Mavingui, Renaud Blonde, Yvonnick Boue, Hamza Berguigua, Jérôme Allyn, Léa Bruneau, Cyril Ferdynus, Nicolas Allou

**Affiliations:** 1 Hôpital Universitaire Félix Guyon, Réanimation Polyvalente, Allée des Topazes, 97400 Saint Denis, France; 2 Hôpital Universitaire Sud Réunion, Réanimation Polyvalente, Avenue François Mitterand BP 350, 97448 Saint Pierre Cedex, France; 3 Laboratoires de Biologie Moléculaire, Hôpital Universitaire Félix Guyon, Allée des Topazes, 97400 Saint Denis, France; 4 UMR Processus Infectieux en Milieu Insulaire Tropical (PIMIT) INSERM 1187, CNRS, Paris, France; 5 Intensive Care Unit, Centre Hospitalier de Mayotte, rue de l’Hôpital, 97600 Mamoudzou, Mayotte France; 6 Département d’Informatique Clinique, Hôpital Universitaire Félix Guyon, Allée des Topazes, 97400 Saint Denis, France; 7 Unité de Soutien Méthodologique, Centre Hospitalier Universitaire Félix Guyon, La Réunion, Saint-Denis, France; 8 INSERM, CIC 1410, Saint-Pierre, France

**Keywords:** Diseases, Virology

## Abstract

The aim of this study was to compare the prognosis of patients with acute respiratory failure (ARF) due to the severe acute respiratory syndrome coronavirus 2 (SARS-CoV-2) variant 501Y.V2 to that of patients with ARF due to the original strain. This retrospective matched cohort study included all consecutive patients who were hospitalized for ARF due to SARS-CoV-2 in Reunion Island University Hospital between March 2020 and March 2021. Twenty-eight in hospital mortality was evaluated before and after matching. A total of 218 patients with ARF due to SARS-CoV-2 were enrolled in the study. Of these, 83 (38.1%) were infected with the 501Y.V2 variant. During intensive care unit stay, 104 (47.7%) patients received invasive mechanical ventilation and 20 (9.2%) patients were supported by venovenous extracorporeal membrane oxygenation. Patients infected with the 501Y.V2 variant were younger (58 [51–68] vs. 67 [56–74] years old, *P* = 0.003), had less hypertension (54.2% vs 68.1%, *P* = 0.04), and had less chronic kidney disease (13.3% vs. 31.9%, *P* = 0.002) than patients infected with the original strain. After controlling for confounding variables (62 matched patients in each group), 28-day mortality was higher in the group of patients infected with the 501Y.V2 variant (30.6%) than in the group of patients infected with the original strain (19.4%, *P* = 0.04). In Reunion Island, where SARS-CoV-2 incidence remained low until February 2021 and the health care system was never saturated, mortality was higher in patients with ARF infected with the 501Y.V2 variant than in patients infected with the original strain.

## Introduction

An outbreak of severe acute respiratory syndrome coronavirus 2 (SARS-CoV-2) that started in China in December 2019 began to spread globally in January 2020^1^. Reunion Island (845,000 inhabitants), a French overseas department located in the Indian Ocean, was relatively spared by the SARS-CoV-2 pandemic until February 2021^2^. From the first detected case of SARS-CoV-2 infection on 19 March 2020 until 4 February 2021, only 10,330 cases and 5,4 deaths per 100,000 inhabitants were reported on the island^2^. These figures are likely due to the protective effects of climatic and environmental factors against SARS-CoV-2 transmission^3–5^ and to the geographical characteristics of Reunion Island (i.e. an insular territory with the international airport as its only entry point). To this is added the fact the local health care system meets European standards (16 extracorporeal membrane oxygenation supports, coronary angiography, all type of surgeries, etc.), and was therefore able to handle all cases without reaching saturation.

In recent months, several SARS-CoV-2 variants of concern have been spreading worldwide. These are a source of worry as they may lead to: reinfection of SARS-CoV-2 recovered individuals^6^; lower effectiveness of vaccines^7,8^; a higher transmission rate^9^; and more severe pathogenicity^9,10^. The 501Y.V2 variant, which has three mutations to the spike protein, first appeared in the Eastern Cape Province of South Africa in October 2020 and then spread to other countries^11,12^. In Reunion Island, the first case of the 501Y.V2 variant was isolated on 4 January 2021 in a patient transferred from the Comoros for acute respiratory failure (ARF). Since then, the incidence of SARS-CoV-2 infection has increased threefold on the island^2^. In Mayotte, another French overseas department in the Indian Ocean, the incidence has increased 17-fold since the 501Y.V2 variant was first detected in January^2,13^. The 501Y.V2 variant is now the most common variant in Reunion Island, Mayotte, and the neighboring islands of the Comoros archipelago^2,13^. At present, no clinical data are available on the pathogenicity of ARF due to SARS-CoV-2 variant 501Y.V2. The aim of this study was to compare the prognosis of patients with ARF due to the 501Y.V2 variant to that of patients with ARF due to the original strain.

## Methods

All methods were performed in accordance with the French legislation on non-interventional studies. This study was registered with the National Institute of Health Data under the number MR4-04 (2206739) and approved by the Ethics Committee of the French Society of Infectious Disease and Tropical Medicine (CER-MIT 2021-N°00011642). Written and oral Informed Consent was obtained from all participants after they were given a written information notice about the process of data collection. All methods were performed in accordance with the relevant guidelines and regulations. This study complies with the Strengthening the Reporting of Observational studies in Epidemiology recommendations statement^14^.

### Selection of the study sample

All consecutive patients with ARF due to SARS-CoV-2 who were hospitalized in one of the three intensive care units (ICUs) of Reunion Island University Hospital between 1 March 2020 and 18 April 2021 were included in the study ((Félix Guyon University Hospital, Saint-Pierre University hospital and Saint-Paul Hospital).

Acute respiratory failure was defined as bilateral pulmonary infiltrates on chest X-ray or computed tomography scan and need for high-flow nasal cannula oxygenation or invasive mechanical ventilation.

All patients with a nasopharyngeal or respiratory sample that tested positive for SARS-CoV-2 by real-time reverse transcription-polymerase chain reaction (RT-PCR) targeting the IP2 and IP4 regions and the N gene were evaluated. All positive samples were analyzed using NucliSens easyMAG system (BioMérieux). From 1 January 2021 onwards, positive samples were also analyzed by genome sequencing using Oxford Nanopore technology, as per the Artic Network’s overlapping amplicon protocol^15,16^.

### Therapeutic management

In accordance with our protocol, all patients with ARF due to SARS-CoV-2 were treated with: (1) dexamethasone at a dosage of 6 mg/day for 10 days^17^; (2) deworming with ivermectine or albendazole; and (3) enhanced anticoagulation, as per the guidelines of the French Society of Thrombosis and Hemostasis and the French Society of Anesthesia and Intensive Care^18^.

High-flow nasal cannula oxygenation was initiated in patients requiring standard oxygen ≥ 9 L/min to maintain peripheral arterial oxygenation saturation ≥ 92%. The timing of intubation and mechanical ventilation was not protocolized but determined by the ICU team on a case-by-case basis.

The exclusion criteria were: too high cycle threshold value in RT-PCR assay for variant screening; ARF due to 501Y.V1; and ARF due to 501Y.V3.

### Data collection and study outcomes

Information was collected on the following: demographic characteristics; comorbidities; organ failure during ICU stay requiring venovenous extracorporeal membrane oxygenation, renal replacement therapy, invasive or non-invasive mechanical ventilation, use of catecholamines; prognosis (mechanical ventilation duration, length of stay in hospital and in ICU, and in-hospital and in-ICU mortality); and morbidity (coinfection, thromboembolic complications, and hospital-acquired pneumonia).

The primary outcome was 28-day in-hospital mortality.

The secondary outcomes were the occurrence of pulmonary embolism, the occurrence of hospital-acquired pneumonia, the need for venovenous extracorporeal membrane oxygenation support, and in-ICU length of stay.

### Statistical analysis

Categorical variables were expressed as total number (percentages). Continuous variables were expressed as median [25th–75th percentiles]. The study cohort was divided into patients infected with the 501Y.V2 variant and patients infected with the original strain. As the study was not randomized, unbalanced covariates could have introduced selection and confusion biases. Moreover, the number of covariates was large relative to the number of primary outcomes. These two problems were addressed by using a matching process based on a propensity score and a prognostic score in which one patient infected with the 501Y.V2 variant was matched with one patient infected with the original strain^19,20^. The propensity score was determined by fitting a logistic regression to estimate the probability of being infected with the 501Y.V2 variant^21^. The prognostic score was determined by fitting a logistic regression to estimate the probability of the primary outcome occurring in patients infected with the original strain (and unlikely to be infected with the 501Y.V2 variant since they were hospitalized between 13 March 2020 and 31 December 2020), and then by applying the generated model to the entire cohort^22^. Patients were matched based on the two scores using a Mahalanobis distance with a caliper width of 0.5^19,20,23^. No replacement was allowed, and all patients were matched only once.

The propensity model was fitted with 2 covariates: patients evacuated by air from Mayotte to Reunion Island and age. The disease risk score model was fitted on the unexposed group with covariates known to be associated with poor prognosis of COVID-19: age, sex, chronic kidney disease, hypertension, diabetes mellitus, congestive heart failure and body mass index > 25 kg/m^2^. Patients were then matched on both scores*.* Baseline characteristics were compared before and after matching. Quantitative variables were compared using the Student’s t-test or Mann–Whitney U test, as appropriate. Qualitative variables were compared using the chi-square test or Fisher’s exact test, as appropriate. The marginal effect of being infected with the 501Y.V2 variant on the primary outcome (with 95% confidence interval) was estimated by applying the Doubly Robust Matching Estimator (DRME) on the matched cohort with proper control of confounding^20^. The advantage of this approach being that only one of the two score models needs to be correct to obtain a consistent estimator^20^. Lastly, the odds ratios of the primary and secondary outcomes (with 95% confidence intervals) were estimated using a conditional logistic regression. A *P-*value < 0.05 was considered significant. All analyses were performed at a two-tailed alpha level of 0.05. Statistical analyses were conducted with SAS 9.4 (SAS Institute, Cary, NC).

### Ethics approval, consent to participate and consent for publication

The present observational study was approved by the Ethics Committee of the French Society of Infectious Disease and Tropical Medicine (CER-MIT 2021-N°00011642) and was declared to the Commission nationale de l’informatique et des libertés (French Data Protection Agency or CNIL MR004). All patients or their legally authorised representative were verbally informed and a written information notice was given about the process of data collection, for publication and could refuse to participate in the study.

## Results

**Figure 1:**
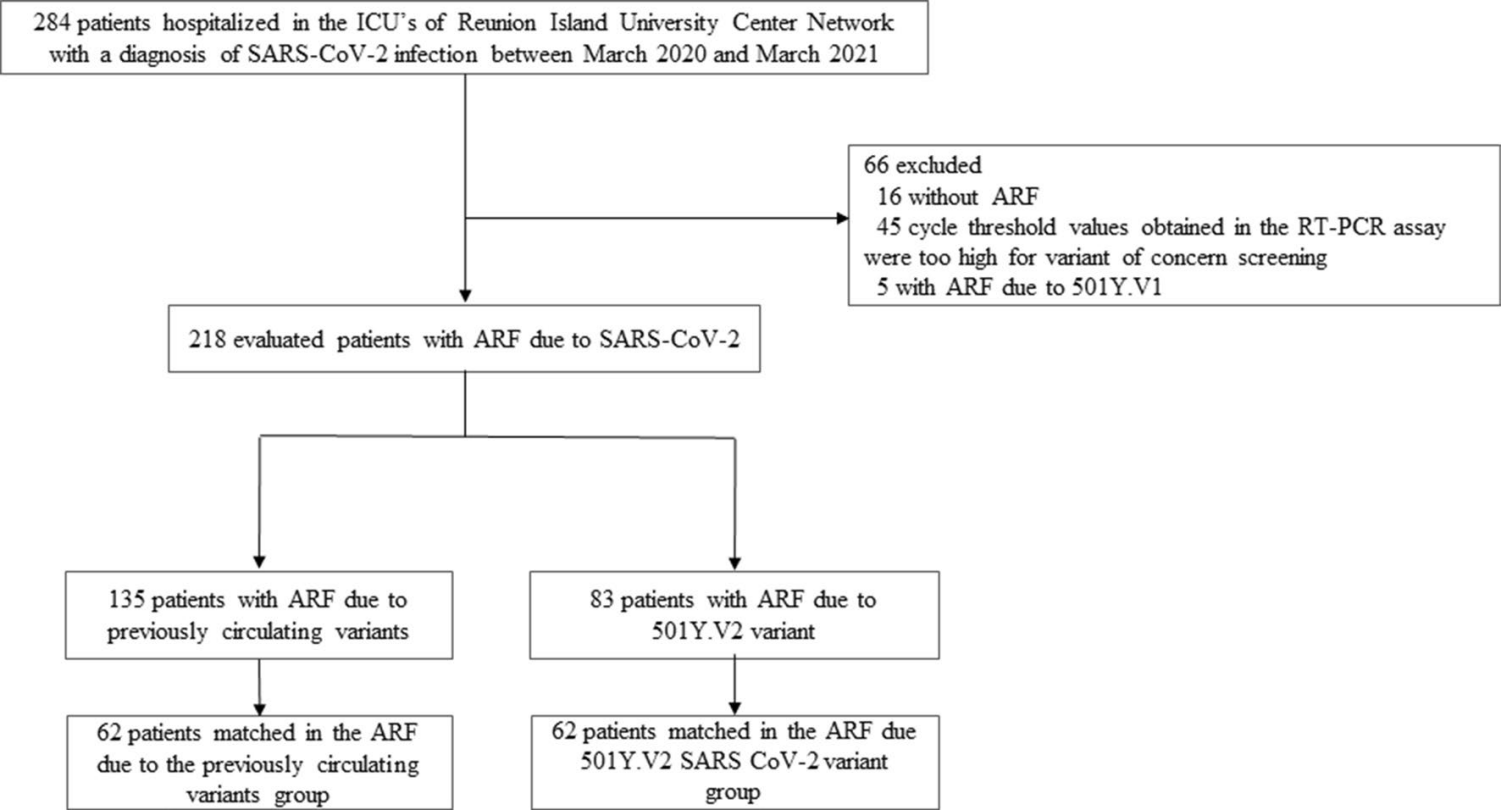
Selection of the study sample.

Over the study period, 284 patients tested positive for SARS-CoV-2 were hospitalized in one of the two ICUs of Reunion Island University Hospital. Of these, 66 were excluded: 16 because they did not develop ARF, 5 had ARF due to 501Y.V1 variant and 45 because the cycle threshold values obtained in the RT-PCR assay were too high for variant of concern screening. The remaining 218 patients formed the cohort (Fig. 1).

Matching resulted in two well-matched groups of 62 patients each (Fig. 1).

### Characteristics of the 218 pre-matched patients

Of the 218 pre-matched patients, 50 (22.9%) were transferred to Reunion Island from Mayotte or the Comoros. Patient characteristics on ICU admission are shown in Table 1. In summary, 83 (38.1%) patients had ARF due to the 501Y.V2 variant. The median Simplified Acute Physiology Score was 32 [25–43], and the median number of days between the onset of symptoms and hospitalization in ICU was 8 [5–11] day. Patients infected with the 501Y.V2 variant were younger (58 [51–68] vs. 67 [56–74] years old, *P* = 0.003), had less hypertension (54.2% vs 68.1%, *P* = 0.04), and had less chronic kidney disease (13.3% vs. 31.9%, *P* = 0.002) than patients infected with the original strain (Table 1).

**Table 1:** Baseline patient characteristics in pre-matched groups.

Variables	Total	501Y.V2		Standardized difference
	(n = 218)	No (n = 135)	Yes (n = 83)	
Patients evacuated by air from Mayotte to Reunion Island	50 (22.9)	13 (9.6%)	37 (44.6%)	0.85
Delay between hospital admission and onset of symptoms (days)	5 [2.5–7]	5 [2–80]	5 [3–7]	0.15
Delay between in ICU admission and onset of symptoms (days)	8 [5–11]	8 [5–11]	7 [5–11]	0.08
**Comorbidities**				
Age (years old)	62 [53–73]	67 [56–74]	58 [51–68]	− 0.42
Male sex	139 (63.8)	81 (60)	58 (69.9)	0.21
Body mass index > 25 kg/m^2^	163 (74.8)	102 (75.6)	61 (73.5)	− 0.05
Cancer (< 3 months)	5 (2.3)	4 (3)	1 (1.2)	− 0.12
History of congestive heart failure	37 (17)	26 (19.3)	11 (11.3)	− 0.16
Diabetes mellitus	95 (43.6)	62 (45.9)	33 (39.8)	− 0.12
Hypertension	137 (62.8)	92 (68.1)	45 (54.2)	− 0.29
Chronic kidney disease	54 (24.8)	43 (31.9)	11 (13.3)	− 0.45
Chronic obstructive pulmonary disease	34 (15.6)	24 (17.8)	10 (12)	− 0.16
Immunodepression	19 (8.7)	13 (9.6)	6 (7.2)	− 0.09
**During the first 24 h in ICU**				
Simplified Acute Physiology Score 2	32 [25–43]	34 [26–45]	31 [24–38]	− 0.23
High− flow oxygen therapy	194 (89)	125 (92.6)	69 (83.3)	− 0.29
Extent of lesions on initial CT scan > 50%	100 (45.9)	59 (45)	41 (49.4)	0.03
*Severe ARDS	49 (22.5)	27 (20)	22 (26.5)	0.20
Glasgow coma scale score	15 [15–15]	15 [15–15]	15 [15–15]	− 0.14
Enhanced thromboprophylaxis	215 (98.6)	132 (97.8)	83 (100)	0.21
Corticosteroids	185 (95.4)	116 (94.3)	69 (97.2)	0.14
Deworming with ivermectine	55 (27.9)	28 (23.1)	27 (35.5)	0.27
C− reactive protein (mg/L)	109 [69–177]	123 [73–187]	90 [68–169]	− 0.08
Creatinin (µmol/L)	93 [66–160]	95 [65–173]	90 [66–126]	− 0.32
D− dimer level (µg/mL)	1085 [609–2210]	1037 [602–1790]	1153 [693–3409]	0.15
Total bilirubin level (mg/dL)	12 [8–16]	12 [8–16]	14 [7–20]	− 0.10
Lactate dehydrogenase (IU/L)	470 [374–605]	498 [386–607]	444 [369–539]	− 0.28
Lymphocytes count (G/L)	0.8 [0.48–1.17]	0.8 [0.5–1.18]	0.78 [0.44–1.05]	− 0.14
Polynuclear neutrophils (G/L)	6.57 [4.33–9.76]	6.47 [4.54–8.98]	6.86 [4.13–10.59]	0.14
Fibrinogen (g/L)	6.1 [5.1—7.1]	6.1 [5.1–7.1]	5.9 [5.1—7.2]	0.35

Results are expressed as total numbers (percentages) for categorical variables and as medians [25th–75th percentiles] for continuous variables as appropriate.

*ARDS* acute respiratory distress syndrome, *CT* computed tomography, *ICU* intensive care unit.

*Defined as PaO2/FIO2 ratio ≤ 100 mmHg with PEEP ≥ 5 cm H2O.

Computed tomography scan showed severe pulmonary involvement (i.e. > 50%) in 100 (45.9%) patients, with no difference between the two groups (*P* = 0.48).

A total of 194 (89%) patients received high-flow nasal cannula oxygenation on ICU admission. Forty-nine (22.5%) patients developed severe acute respiratory distress syndrome in the first 24 h following admission. During ICU stay, 104 (47.7%) patients were intubated, of whom 101 (97.1%) received continuous neuromuscular blockade, 76 (73.1%) received prone position therapy, and 27 (26%) received nitric oxide. Moreover, 42 (19.3%) patients presented with acute kidney failure requiring renal replacement therapy, and 83 (38.1%) patients were treated with norepinephrine.

Patients infected with the 501Y.V2 variant had a 28-day in-hospital mortality of 32.5%, compared to 22.2% for patients infected with the original strain (*P* = 0.1).

### Characteristics and prognosis of the 124 matched patients

After matching, there were no significant differences in characteristics between the group of patients infected with the 501Y.V2 variant and the group of patients infected with the original strain (Table 2).

**Table 2:** Baseline patient characteristics in propensity-matched groups.

Variables	Total	501Y.V2 variant		Standardized difference
	(n = 124)	No (n = 62)	Yes (n = 62)	
Patients evacuated by air from Mayotte to Reunion Island	29 (23.4)	13 (21.0)	16 (25.8)	0.11
Delay between hospital admission and onset of symptoms (days)	5.5 [3–8]	6 [3–10]	5 [3–7]	0.01
Delay between in ICU admission and onset of symptoms (days)	8 [5–11]	10 [5–12]	7 [5–10]	− 0.26
**Comorbidities**				
Age (years old)	60 [53–70]	61 [56–70]	60 [53–71]	0.01
Male sex	39 (31.5)	19 (30.6)	20 (32.3)	− 0.03
Body mass index > 25 kg/m^2^	93 (75)	48 (77.4)	45 (72.6)	− 0.11
Cancer (< 3 months)	4 (3.2)	3 (4.8)	1 (1.6)	− 0.18
History of congestive heart failure	21 (16.9)	11 (17.7)	10 (16.1)	− 0.04
Diabetes mellitus	46 (37.1)	24 (38.7)	22 (35.5)	− 0.07
Hypertension	64 (51.6)	32 (51.6)	32 (51.6)	0
Chronic kidney disease	21 (16.9)	10 (16.1)	11 (17.7)	0.04
Chronic obstructive pulmonary disease	16 (12.9)	10 (16.1)	11 (17.7)	0.04
Immunodepression	11 (8.9)	6 (9.7)	5 (8.1)	− 0.06
**During the first 24 h in ICU**				
Simplified Acute Physiology Score 2	31 [24–42]	34 [24–46]	27 [24–37]	− 0.23
High-fow oxygen therapy	109 (87.9)	56 (90.3)	53 (85.5)	− 0.12
Extent of lesions on initial CT scan > 50%	63 56.8)	33 (62.3)	30 (51.7)	− 0.18
*Severe ARDS	32 (25.8)	15 (24.2)	17 (27.4)	0.06
Glasgow coma scale score	15 [15–15]	15 [15–15]	15 [15–15]	− 0.16
Enhanced thromboprophylaxis	124 (100)	62 (100)	62 (100)	0
Corticosteroids	106 (95.5)	53 (94.6)	53 (96.4)	0.08
Deworming with ivermectine	36 (31.9)	16 (29.6)	20 (33.9)	0.09
C-reactive protein (mg/L)	106 [69–171]	117 [73–176]	90 [69–168]	− 0.12
Creatinin (µmol/L)	82 [64–132]	78 [62–124]	87 [68–160]	− 0.12
D-dimer level (µg/mL)	969 [589–1915]	1271 [568–2393]	884 [589–1846]	0.43
Lactate dehydrogenase (IU/L)	448 [395–625]	512 [463–625]	445 [374–541]	− 0.16
Lymphocytes count (G/L)	0.83 [0.51–1.21]	0.8 [0.5–1.14]	0.84 [0.54–1.25]	− 0.17
Polynuclear neutrophils (G/L)	6.22 [4.07–9.97]	6.22 [4.3–10.6]	6.32 [3.97–9.71]	0.04
Fibrinogen (g/L)	6 [5.1–7.12]	6 [5.21–7.08]	6.5 [5.09–7.36]	0.14

*ARDS* acute respiratory distress syndrome, *CT* computed tomography, *ICU* intensive care unit.

Results are expressed as total numbers (percentages) for categorical variables and as medians [25th–75th percentiles] for continuous variables as appropriate.

*Defned as PaO2/FIO2 ratio ≤ 100 mmHg with PEEP ≥ 5 cm H2O.

**Figure 2:**
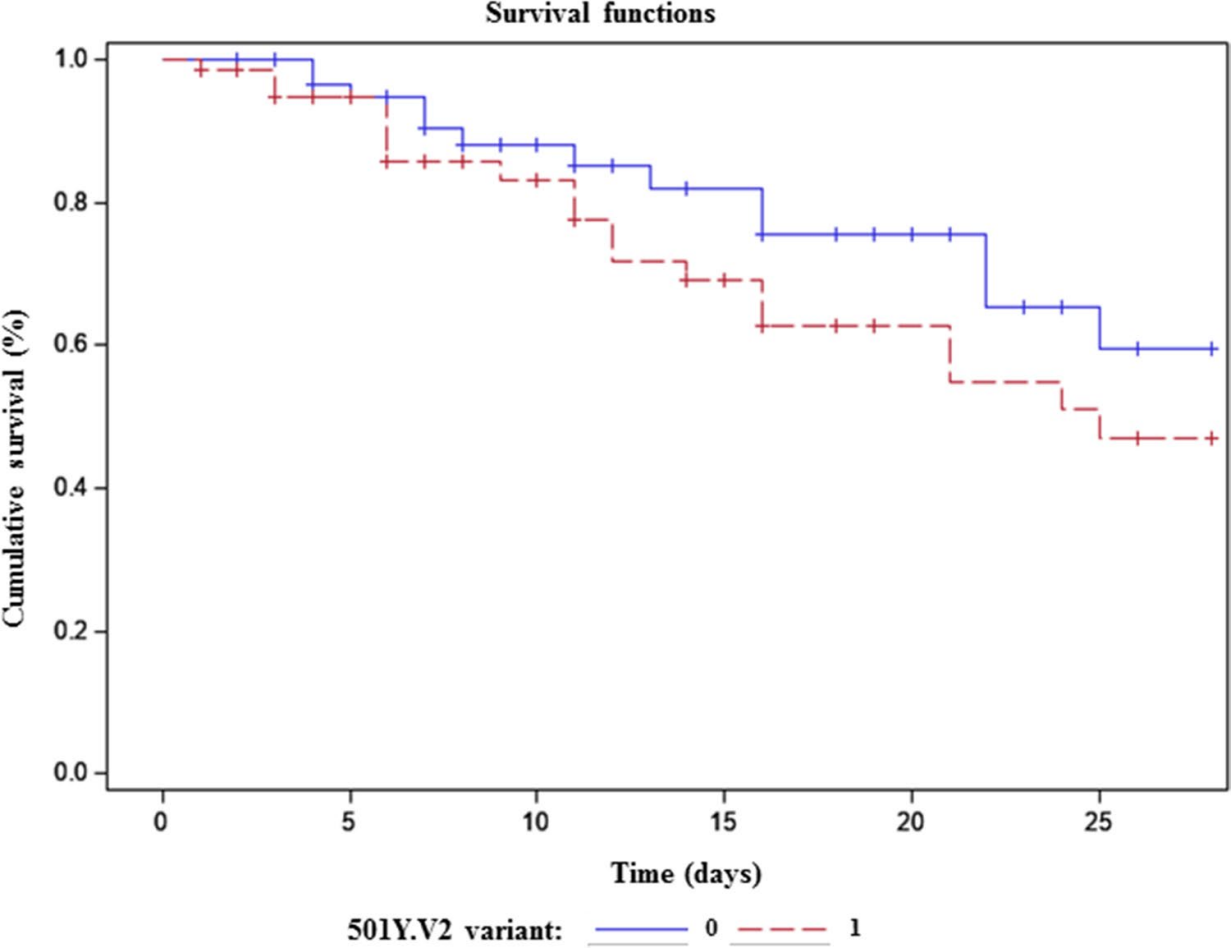
Survival rate for the 124 matched patients (*P* = 0.04).

Patients infected with the 501Y.V2 variant had significantly higher 28-day in-hospital mortality (30.6%) than patients infected with the original strain (19.4%, OR:2.4; 95% CI 1.1–5.8 *P* = 0.04) (Fig. 2) (DRME: 0.12; 95% CI 0.01–0.24).

Pulmonary embolism occurred in 11 (17.7%) patients infected with the 501.YV2 variant and in 2 (3.2%) patients infected with the original strain (*P* = 0.03).

Hospital-acquired pneumonia occurred in 17 (43.6%) patients infected with the 501Y.V2 variant and in 8 (20.5%) patients infected with the original strain (*P* = 0.03) (Table 3).

**Table 3:** Outcome of the 124 matched patients during intensive care unit stay.

Variables	Total	501Y.V2 variant		*OR (95% CI)	*P*-value
	(n = 124)	No (n = 62)	Yes (n = 62)		
Day-28 mortality	31 (25)	12 (19.4)	19 (30.6)	2.4 (1.1–5.8)	0.04
Pulmonary embolism	13 (10.5)	2 (3.2)	11 (17.7)	10 (1.3–78.2)	0.03
Hospital-acquired pneumonia	40 (32.3)	14 (22.6)	26 (41.9)	3 (1.2–7.6)	0.02
Extracorporeal membrane oxygenation	13 (10.5)	4 (6.5)	9 (14.5)	6 (0.7–49.8)	0.1
Renal replacement therapy	21 (16.9)	10 (16.1)	11 (17.7)	1.1 (0.4–3.1)	0.8
Prone position	48 (38.7)	23 (37.1)	25 (40.3)	1.2 (0.5–2.6)	0.68
Continuous neuromuscular blockers	59 (47.6)	27 (43.5)	32 (51.6)	1.6 (0.7–3.6)	0.3
Invasive mechanical ventilation	60 (48.4)	27 (43.5)	33 (53.2)	1.7 (0.7–3.8)	0.41
Nitric oxide	17 (13.7)	9 (14.5)	8 (12.9)	0.6 (0.2–1.9)	0.81
ICU lenght of stay (days)	10 [5–21]	10 [6–21]	9.5 [4–21]	–	0.62

Results are expressed as total numbers (percentages) for categorical variables and as medians [25th–75th percentiles] for continuous variables as appropriate.

*ICU* intensive care unit, *OR* odds ratio, *CI* confidence interval.

*OR and 95%CI were estimated using univariate conditional logistic regression.

Doubly Robust Matching Estimator : 0.12 (95% CI 0.01–0.24).

The need for venovenous extracorporeal membrane oxygenation (*P* = 0.1), and in-ICU length of stay were similar between the two groups (*P* = 0.62) (Table 3).

## Discussion

Our study is the first to compare the prognosis of patients with ARF due to the 501Y.V2 variant to that of patients with ARF due to the original strain. Patients infected with the 501Y.V2 variant had a 28-day in-hospital mortality of 32.5%, compared to 22.2% for patients infected with the original strain. This excess mortality was confirmed after matching for comorbidities and initial severity (30.6% vs. 19.4%, *P* = 0.04).

These findings are unlikely to be the result of inadequate management of SARS-CoV-2 patients in our ICUs. Indeed, the SARS-CoV-2 epidemic took hold later and more slowly in Reunion Island than it did in Europe and the Americas. One consequence of this is that the local health care system, which meets European standards, never reached saturation. Another consequence is that we were able to draw on the experience of other health care centers in the management of SARS-CoV-2 patients. Right from the start, we privileged non-invasive ventilation over invasive ventilation (89% of patients on admission)^24^ extensive use of corticosteroids (95.4% of patients)^17^, and provided enhanced anticoagulation to reduce thromboembolic risk (98.6% of patients)^25^. The adequate management of SARS-CoV-2 patients in Reunionese ICUs is confirmed by the fact that mortality observed in patients infected with the original strain is comparable to mortality rates reported in the recent literature^24,26,27^. Thus, the study by Kurtz et al.^24^ found an in-ICU mortality of 31% in a cohort of 13,301 ICU patients infected with SARS-CoV-2, while that by Schmidt et al. reported a 28-day in-hospital mortality of 30% in a cohort of 4,244 patients^26^. Given these optimal management conditions, the excess mortality observed in our cohort is likely explained by the increased virulence of the 501Y.V2 variant relative to the original strain. This is supported by the fact that the number of recorded deaths due to SARS-CoV2 increased twofold in Reunion Island and threefold in Mayotte since the 501Y.V2 variant was first detected on these islands (January 2021)^2,13^. Likewise, in South Africa, a preliminary analysis performed by the National Institute of Communicable Diseases found that mortality due to SARS-CoV2 was 20% higher during the second wave of the pandemic (when the majority of cases were due the 501Y.V2 variant) than during the first wave^28^. It should be noted that other variants were also found to be more virulent than the original strain^29,30^. In the United Kingdom, a retrospective study found a higher mortality of the 501Y.V1 variant compared to the original strain^30^.

The excess mortality associated with the 501Y.V2 variant is especially worrying considering that our data were obtained when the local health care system was not saturated. As is the case with other SARS-CoV-2 variants, the 501Y.V2 variant appears to be more contagious than the original strain^31–33^. This means that saturation, a known risk factor for mortality^34^, could rapidly occur, with severe forms affecting patients who are younger and have less comorbidities. There is also a risk that the 501Y.V2 variant will undermine the vaccination campaign which is currently one of the pillars of the fight against SARS-CoV-2. The ChAdOx1 nCoV-19 Covid-19 vaccine, so far administered to approximately 3 million people in France, does not protect against medium to moderate infections by the 501Y.V2 variant^8,35^. Similarly, the Novavax vaccine is only 60% effective against this variant^7^, and other vaccines (AZD1222, NVX-CoV237, Ad26.COV2.S and BNT162b2) have shown lower efficacy in South Africa^35^.

In the present study, other arguments that could be in favor of a greater pathogenicity of the 501Y.V2 variant are the higher incidence of pulmonary embolism and hospital-acquired pneumonia in the group of patients infected with the 501Y.V2 than in the group of patients infected with the original strain without significant difference after matching for many comorbidities, corticosteroids use or enhanced thromboprophylaxis.

Our study has several limitations. Biases may have been introduced due to the retrospective nature of the study. In particular, a selection bias may have occurred as a significant proportion of patients infected with the 501Y.V2 variant were transferred from Mayotte, whose population is younger and presents fewer comorbidities than the Reunionese population. As randomization could not be achieved, we used a robust statistical methodology based on matching to minimize this bias^20–23^. Moreover, matching did not completely eliminate the imbalance between groups (the standardized difference remained above 0.1 for some variables)^36^. In addition, mortality due to the 501Y.V2 variant was probably underestimated as several samples could not be screened for variants of concern. Indeed, the patient exclusion rate of 23.2% was relatively high in our study and may represent a selection bias. However, in the study by Challen et al*.* that evaluated the pathogenicity of the 501Y.V1 variant the exclusion rate was 50.7%^30^. While our study sample may seem small (n = 218), it should be noted that all patients hospitalized in our two ICUs for ARF due to SARS-CoV-2 were evaluated, which also helped to reduce the selection bias. Moreover, patient management was the same in both ICUs, and unlike what was the case in many in other studies on the subject, it was optimal throughout the study period^24,26^.

## Conclusion

In Reunion Island, where SARS-CoV-2 incidence remained low until February 2021 and the health care system was never saturated, mortality was higher in patients infected with the 501Y.V2 variant than in patients infected with the original strain. These results are in line with reports on other SARS-CoV-2 variants of concerns, and should be considered in the future management of the pandemic.

## Data Availability

The dataset used in the current study are available from the corresponding author.

## Abbreviations

SARS-CoV-2: Severe acute respiratory syndrome coronavirus 2
ARF: Acute respiratory failure
ICU: Intensive care unit
RT-PCR: Real-time reverse transcription-polymerase chain reaction
DRME: Doubly robust matching estimator
